# Modeling the Invasion: Habitat Suitability and Landscape Connectivity of Wild Boar (*Sus scrofa*) on Jeju Island, South Korea

**DOI:** 10.1002/ece3.74398

**Published:** 2026-09-30

**Authors:** Binod Kunwar, Suraj Baral, Young‐Hun Jeong, Hong‐Shik Oh

**Affiliations:** ^1^ Interdisciplinary Graduate Program in Advanced Convergence Technology and Science Jeju National University Jeju Republic of Korea; ^2^ Herpetology Section Leibniz Institute for the Analysis of Biodiversity Change, Museum Koenig Bonn Bonn Germany; ^3^ Faculty of Science Education Jeju National University Jeju Republic of Korea

**Keywords:** connectivity, conservation, habitat suitability, invasion, *Sus scrofa*

## Abstract

Invasive species pose significant ecological and economic threats to island ecosystems, and identification of suitable habitats and understanding the spread dynamics of invasive species are crucial for effective management. On Jeju Island, South Korea, the invasive wild boar ( *Sus scrofa* ) has rapidly expanded its range, raising ecological and agricultural concerns. This study assessed fine‐scale habitat suitability and landscape connectivity using an optimized Maximum Entropy (maxEnt) model and Linkage Mapper, respectively. After multicollinearity testing, 94 occurrence points and 10 environmental variables were retained for the final model projection. The model demonstrated excellent performance as indicated by high value of the Area Under the Receiver Operating Characteristic Curve (AUC), True Skill Statistics (TSS), and Continuous Boyce Index (CBI). Distance to agriculture, distance to built‐up area, elevation, and distance to water were the most influential predictors of wild boar distribution. The model identified 14.49% of Jeju Island as a highly suitable habitat and 21.64% as suitable, indicating potential expansion and increased human‐wild boar conflict. Connectivity analysis revealed six core habitats linked by eight corridors. Hallasan area and Geumakseo‐gil area were the most important habitats for maintaining overall landscape connectivity. In conclusion, our findings identified potential risk areas, prioritized targeted monitoring, early detection, and proactive management to limit future expansion and reduce the ecological and agricultural impacts of wild boar on Jeju Island.

## Graphical abstract

The integration of habitat suitability modeling and landscape‐level connectivity analysis for invasive species; wild boar, at fine‐scale resolution provides a comprehensive framework for identifying potential spatial distribution, dispersal corridors, and assessing expansion dynamics. Findings identified potential risk areas, prioritized targeted monitoring, early detection, and proactive management to limit future expansion and reduce the ecological and agricultural impacts of wild boar on Jeju Island.

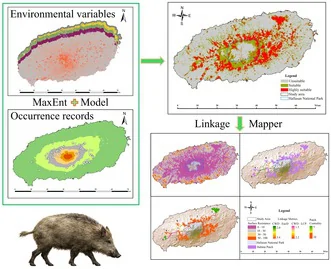

## 1 Introduction

Island biodiversity is highly vulnerable to climate change and biological invasions (Spatz et al. 2017). Terrestrial mammal invasive species are the primary drivers of island native species extinctions (Medina et al. 2011), where their impacts are often more severe than on continental ecosystems due to ecological isolation, lack of predator defense mechanisms, and high endemism (Blumstein and Daniel 2005; Kier et al. 2009; Bodey et al. 2023). Identifying invasive species distribution patterns and habitat connectivity is essential for effective conservation management (Stewart‐Koster et al. 2015; Kang et al. 2022).

The wild boar ( *Sus scrofa* ; Mammalia; Suidae) is native to Eurasia and a widely spread mammal species (Barrios‐Garcia and Ballari 2012). Wild boar as an invasive species poses serious environmental and economic concerns, such as crop damage (Lee and Lee 2019), rooting (Stoakley et al. 2025), vehicle collision (Beasley et al. 2014), disease transmission (Han, et al. 2022), and hybridization (Iacolina et al. 2018). Originally a native species, the wild boar became extinct over time on Jeju Island in South Korea. The genetic analysis of the present‐day population showed that Jeju wild boars are more closely associated with the Chinese population than the mainland Korea (Choi et al. 2014). On Jeju Island, wild boars were deliberately released or escaped from farms in 2000. Although the wild boar population size remains undocumented, they have established a self‐sustaining wild population on the island (Jo et al. 2012). The wild boar poses significant ecological and agricultural impacts and is classified as an invasive species on Jeju Island. To mitigate these impacts, the Ministry of Environment, South Korea has implemented culling programs; however, these measures have not resulted in sustained long‐term population control, with the wild boar continuing to persist across the island (MoE 2014). Therefore, modeling of habitat suitability and landscape‐level connectivity is necessary for informing effective management decisions and strategic conservation planning.

Species distribution models (SDMs) are widely used in invasion ecology to predict potential geographical expansion of invasive species (Lewis et al. 2017; Gao et al. 2025). Among these, the Maximum Entropy model (MaxEnt) is widely applied for its robust predictive performance with species presence‐only data (Phillips et al. 2006; Elith et al. 2010). Invasive species distribution modelling is crucial for early detection, identification of high‐risk areas, prioritization of monitoring efforts, and efficient allocation of management resources (Reaser et al. 2020; Shabani et al. 2020). Habitat connectivity facilitates or constrains movement across the landscape, thereby shaping dispersal dynamics (Glen et al. 2013; Kramer et al. 2024). Integration of habitat suitability modeling and landscape‐level connectivity at fine‐scale resolution provides a comprehensive framework for assessing potential spatial distribution, dispersal corridors, and expansion, supporting effective and proactive invasive species management and conservation planning (Descombes et al. 2016; Faustini et al. 2025).

In this study, we assessed the effects of environmental variables on the habitat suitability and landscape connectivity of wild boar on Jeju Island, South Korea, with the objective of predicting habitat suitability and identifying suitable habitat patches. In addition, we explored the least‐cost dispersal pathways and potential corridors connecting core habitats. This study aimed to establish a scientific foundation for strategic management and control of wild boar on Jeju Island.

## 2 Materials and Methods

### 2.1 Study Area

This study was conducted on Jeju Island (33°11′–33°33′ N, 126°08′–126°58′ E); the largest volcanic island in the southern Korean Peninsula (Figure 1), encompassing the highest mountain Hallasan (1950 m a.s.l), steep hills, oreums (parasitic volcanic cones), lava tubes (volcanic caves), valleys, and coastal plains (Woo et al. 2013). The island has a temperate climate with most rainfall between April and July, and daily high temperature ranges from 15.5°C to 24.4°C (KMA 2024). Hallasan National Park exhibits unique vertical ecosystems on the island. The island is free of large predator and supports 28 species of terrestrial mammals (Jo et al. 2012). The terrestrial mammals include four invasive species: sika deer ( *Cervus nippon* ), red deer ( *Cervus elaphus* ), wild boar ( *Sus scrofa* ), and siberian chipmunk ( *Tamias sibiricus* ) (Jo et al. 2012). The study area includes 9.19% of built‐up areas, 35.35% of agriculture, 33.49% of forest, 18.80% of grassland, 2.52% of bare land, and 0.65% of water bodies in 2024 (MoE 2025).

**FIGURE 1:**
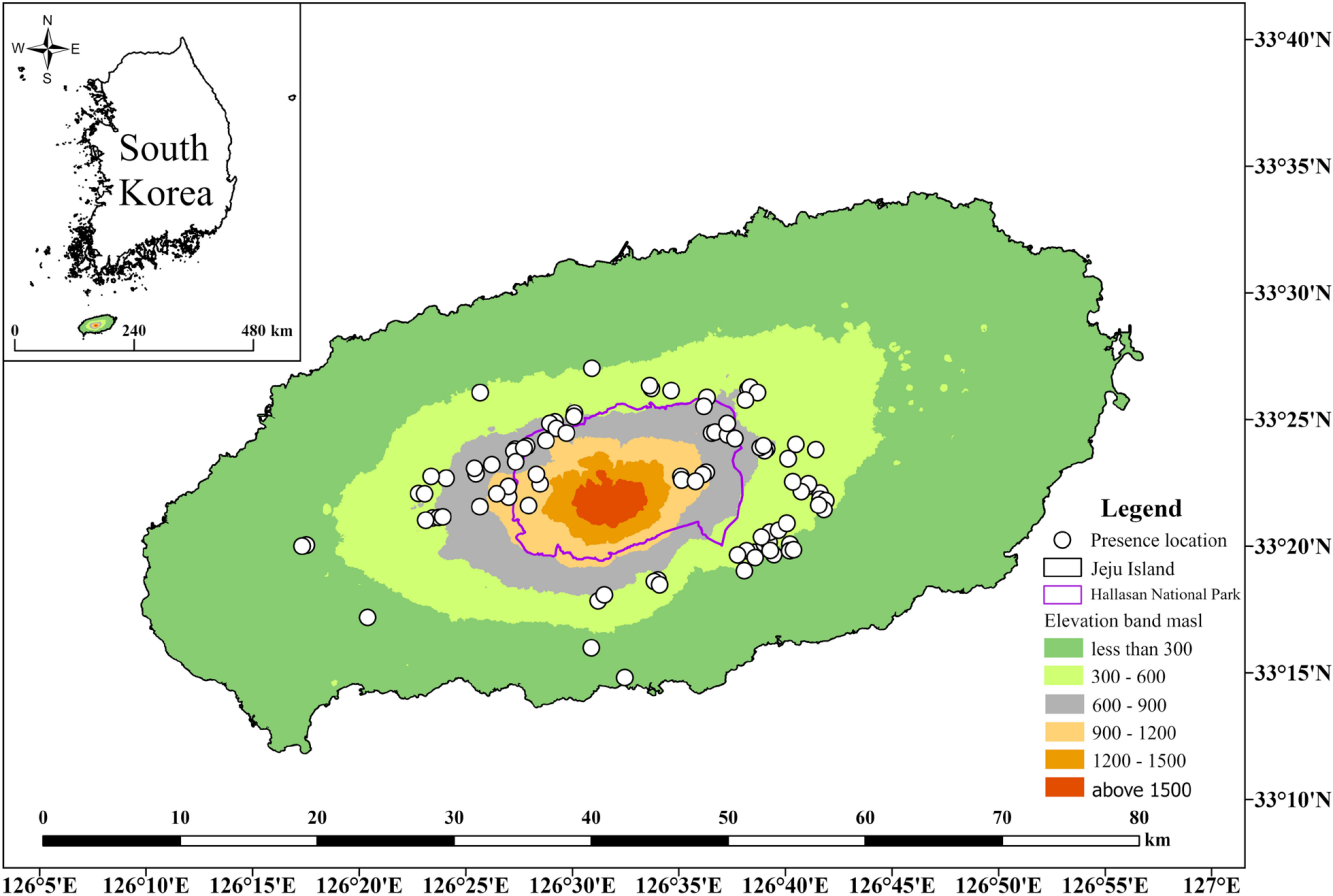
Elevation bands and occurrence points of wild boar for the MaxEnt model on Jeju Island and South Korea (inset).

### 2.2 Species Occurrence Data

Species occurrence data were compiled from opportunistic sightings and images from camera traps laid during field surveys from 2023 to 2025. Jeju Island was divided into 1 × 1 km grids, and camera traps were deployed within each grid during the annual field survey. Based on annual field survey data and field signs such as rubbing trees, feces, wallowing, and soil rooting, potential wild boar sites were identified for camera trap installments. A total of 120 camera traps (ROBOT D30, Bushwhacker, China) were deployed at least 30 m from each other for at least a month. Camera traps had an aperture angle of 90° and movement detection by passive infrared sensors (PIR) with a detection range of ≤ 20 m. Camera traps were programmed in hybrid mode and captured a burst of three photographs (20MP) followed by 30 s video (resolution: 1920 × 1080 pixels) upon each trigger. A total of 94 georeferenced wild boar occurrences were utilized after spatial rarefaction in SDMtoolbox Pro 0.9.1 employed in ArcGIS Pro 3.2.0 (Boria et al. 2014; Esri 2023) (Table S1). Of these 85 occurrence records from camera trapping images, the remaining records were derived from opportunistic sightings (Table S1). Only one occurrence was retained per 30 × 30 m grid to evaluate fine‐resolution habitat suitability (Figure 1).

### 2.3 Environmental Predictors

Our study included 14 selected environmental variables (Table S2). All variables were analyzed in the UTM 52 N projection (WGS‐1984) and resampled to a spatial resolution of 30 m (Tables 1 and S2). The land use and land cover (LULC) 2025 with a spatial resolution of 1 m was downloaded from the Ministry of Environment, South Korea (MoE 2025). The Euclidean distances to built‐up area, agriculture, forest, grassland, barren land, and water were extracted from LULC in ArcGIS Pro. Habitat heterogeneity was computed using the “Focal Statistic” tool in ArcGIS Pro within a 33 × 33 moving window from LULC to represent the daily movement of wild boar (Lee, Kim, et al. 2024). A Digital Elevation Model (DEM) was downloaded from the Shuttle Radar Topography Mission (SRTM 2013), and then the “Spatial Analyst” tool was utilized to derive slope and terrain ruggedness index (TRI) from the DEM. The annual median values from 2020 to 2025 for the normalized difference vegetation index (NDVI), normalized difference built‐up index (NDBI), and modified normalized difference water index (MNDWI) were retrieved from the Google Earth Engine (GEE) (Gorelick et al. 2017). The NDVI is used to quantify vegetation greenness, density, and overall vegetation condition (Xu and Guo 2014), whereas NDBI is used to quantify built‐up or developed areas condition (Zha et al. 2003), and MNDWI detects and enhances open water features (Xu 2006). NDVI, NDBI, and MNDWI were calculated from Landsat 8 imagery at a resolution of 30 m using the red and near‐infrared (NIR) bands (Equation 1), the shortwave‐infrared (SWIR) and NIR bands (Equation 2), and the green and SWIR bands (Equation 3), respectively.

(1) $$\text{NDVI}=\frac{\left(\mathrm{NIR}-\mathrm{Red}\right)}{\left(\mathrm{NIR}+\mathrm{Red}\right)}\;\text{or}\;\frac{\left(\text{Band}\;5-\text{Band}\;4\right)}{\left(\text{Band}\;5+\text{Band}\;4\right)}$$

(2) $$\text{NDBI}=\frac{\left(\text{SWIR}-\mathrm{NIR}\right)}{\left(\text{SWIR}+\mathrm{NIR}\right)}\;\text{or}\;\frac{\left(\text{Band}\;6-\text{Band}\;5\right)}{\left(\text{Band}\;6+\text{Band}\;5\right)}$$

(3) $$\text{MNDWI}=\frac{\left(\text{Green}-\text{SWIR}\right)}{\left(\text{Green}+\text{SWIR}\right)}\;\text{or}\;\frac{\left(\text{Band}\;3-\text{Band}\;6\right)}{\left(\text{Band}\;3+\text{Band}\;6\right)}$$

**TABLE 1:** Environmental variables selected for the final wild boar distribution model.

Variable	Variable code	Description	Unit	Original resolution (m)	Source
Distance to built‐up area	dist_built	Euclidean distance to built‐up Area	m	30	Land use and land cover of 2024 (https://egis.me.go.kr/)
Distance to agriculture	dist_agri	Euclidean distance to agriculture	m	30	Land use and land cover of 2024 (https://egis.me.go.kr/)
Distance to forest	dist_forest	Euclidean distance to forest	m	30	Land use and land cover of 2024 (https://egis.me.go.kr/)
Distance to barren land	dist_bare	Euclidean distance to barren land	m	30	Land use and land cover of 2024 (https://egis.me.go.kr/)
Distance to water resources	dist_water	Euclidean distance to water resources	m	30	Land use and cover of 2024 (https://egis.me.go.kr/)
Elevation	alt	Elevation above sea level	m	30	https://www.earthdata.nasa.gov/
Slope	slope	Gradient of slope	°	30	Digital Elevation Model
Terrain ruggedness index	tri	Topographic heterogeneity	—	30	Digital Elevation Model
Normalized difference vegetation index	ndvi	Near infrared bands and red bands used from Landsat 8 imagery to qualify vegetation	—	30	https://earthengine.google.com/
Modified normalized difference water index	mndwi	Green and shortwave‐infrared bands used from Landsat 8 imagery to detect and identify open water	—	30	https://earthengine.google.com/

To test multicollinearity, a Spearman's rank correlation analysis was conducted and uncorrelated variables (|r| ≤ 0.70) were used for the final distribution projection (Naimi and Araújo 2016). Consequently, 10 environmental variables were selected for the final wild boar distribution model (Table 1, Figure S1).

### 2.4 Modeling Habitat Suitability

Maximum Entropy (MaxEnt) version 3.4.0 (Phillips et al. 2006) was used to model wild boar habitat suitability in the study area. A Gaussian kernel density was generated from occurrence records to represent spatial variation in sampling intensity. This density was used to probabilistically generate 10,000 bias‐weighted background points, which were subsequently used as the fixed background sample in the MaxEnt model. To overcome the complexity or overfitting through the default parameters of the MaxEnt model, tuning of key parameters was done that enhances the model accuracy (Phillips and Dudík 2008). The possible combinations of feature classes (linear, quadratic, hinge, and product) and beta multipliers ranging from 0.5 to 5 with an increment of 0.5, were analyzed (Figure S2). After a comprehensive assessment, the model with the optimized beta multiplier and combined feature class yielding the lowest Akaike Information Criterion value (delta AICc = 0) was the best fit and selected for the final projection (Huang et al. 2019). The AICc‐based approach minimizes overfitting risk and achieves the balance between goodness‐of‐fit and model complexity, enhancing the habitat suitability prediction's reliability. Model parameter optimization was done with the “ENMeval” package (Kass et al. 2021) in R version 3.2.2 (R Core Team 2016).

After optimizing the model parameters, the final MaxEnt model was run with 10 replicates with subsampling, with both occurrence and background data split into 70% for training and 30% for testing, implemented in R using “Biomod2” package (Thuiller et al. 2016). The model performance was evaluated using Area Under the Receiver Operating Characteristic Curve (AUC) (Fielding and Bell 1997), True Skill Statistics (TSS) (Allouche et al. 2006), and Continuous Boyce Index (CBI) (Hirzel et al. 2006). The variable importance was calculated by a permutation‐based method in “Biomod2”. The response curve was generated by an in‐built function that adopts the evaluation strip method in “Biomod2” (Thuiller et al. 2009). The resulting habitat suitability, with a probability ranging between 0 and 1, was categorized into unsuitable and suitable areas based on the maximum training sensitivity plus specificity approach (Liu et al. 2016). The threshold was calculated as 0.38, and the area with habitat suitability above this threshold was further classified into suitable (0.38–0.6) and highly suitable (0.6–1) categories.

### 2.5 Analysis of Habitat Patches and Connectivity

The habitat suitability map from the final model was used to prepare a landscape resistance model. The landscape resistance was computed from a formula adopted from Keeley et al. (2016):

$$R=100-99\times \frac{\left(1-\exp\left(-c\times H\right)\right)}{\left(1-\exp\left(-c\right)\right)}$$

where *R* denotes resistance, *H* represents habitat suitability, *exp* is the exponential function, and *c* is the transformation threshold which was set to a value 8 for optimal performance (Keeley et al. 2016; Niyogi et al. 2022). This moderate threshold was selected as appropriate for species exhibiting ecological plasticity (Keeley et al. 2016; Johann et al. 2020).

Habitat patches were delineated as patches with a suitability score above 0.6 (highly suitable pixels) and larger than 3 km^2^ based on the home range of wild boar (Fattebert et al. 2017; Schlichting et al. 2022; Lee, Kim, et al. 2024). Habitat patches separated by less than or equal to 1 km were merged using the “Aggregate Polygons” tool in ArcGIS Pro based on the daily movement of wild boar (Lee, Song, and Choi 2024). The movement of wild boar on Jeju Island was analyzed by Least‐Cost Pathways (LCP). The least‐cost pathways (LCP) algorithms were used for landscape‐level connectivity of habitat patches in the Linkage Mapper 3.1.0 tool, employing the “Build network and map linkages” function in ArcGIS Pro (McRae and Kavanagh 2011). Relative connectivity assessment was evaluated using two metrics: the ratio of cost‐weighted distance (CWD) to Euclidean distance and the ratio of cost‐weighted distance (CWD) and least‐cost pathway (LCP), where lower ratios of the two metrics indicate easier linkage and offer higher connectivity (Dutta et al. 2016). Then, the importance of each habitat patch was evaluated using the “Centrality mapper” function of the Linkage Mapper tool within ArcGIS Pro. Centrality analysis measures the contribution of each habitat patch to maintain the overall landscape connectivity. Higher centrality scores indicate greater accessibility within the landscape (Carroll et al. 2012).

## 3 Results

### 3.1 Model Performance and Variable Importance

The final MaxEnt model included linear, quadratic, and hinge features with beta multiplier value of 2.5 (Figure S2). Predictive performance of the best model was high, with an AUC value of 0.76 ± 0.05, a TSS value of 0.46 ± 0.04, and a CBI value of 0.82 ± 0.08.

Among the 10 environmental variables incorporated into the final MaxEnt model, the distance to agriculture (0.64), followed by the distance to built‐up area (0.22), the elevation (0.14), and the distance to water (0.13) were the most important predictors for defining wild boar distribution on Jeju Island (Figure 2 and Table S3). The remaining predictors: MNDWI, slope, the distance to forest, TRI, NDVI, and the distance to bare land were less influential variables in shaping wild boar distribution (Figure 2 and Table S3).

**FIGURE 2:**
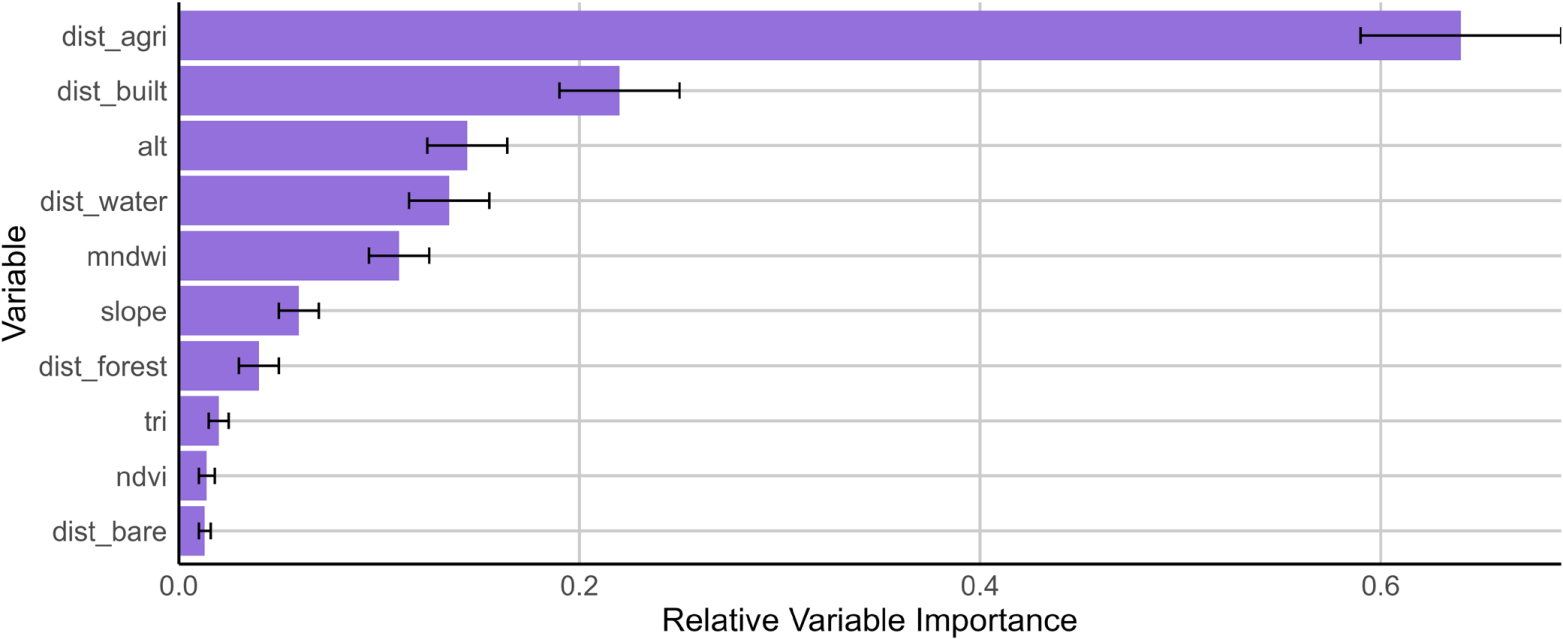
Relative importance of analyzed variables; dist_agri: Euclidean distance to agriculture, dist_built: Euclidean distance to built‐up area, alt: Elevation, dist_water: Euclidean distance to water, mndwi: Mean normalized difference water index, slope, dist_forest: Euclidean distance to forest, tri: Terrain ruggedness index, ndvi: Normalized difference vegetation index, and dist_bare: Euclidean distance to bare land in the final MaxEnt Model for distribution of wild boar on Jeju Island.

### 3.2 Species Response Curve

Wild boar exhibited a strong preference for elevation from 0 to 500 m.a.s.l., and suitability increased sharply with the distance from the agricultural landscape up to 500 m and then remained relatively stable (Figure 3). Wild boar showed a positive association with distance to agricultural landscape, built‐up area, MNDWI, slope, distance to forest, and NDVI (0.4–0.7). In contrast, wild boar showed a negative association with TRI and distance to bare land (Figure S3).

**FIGURE 3:**
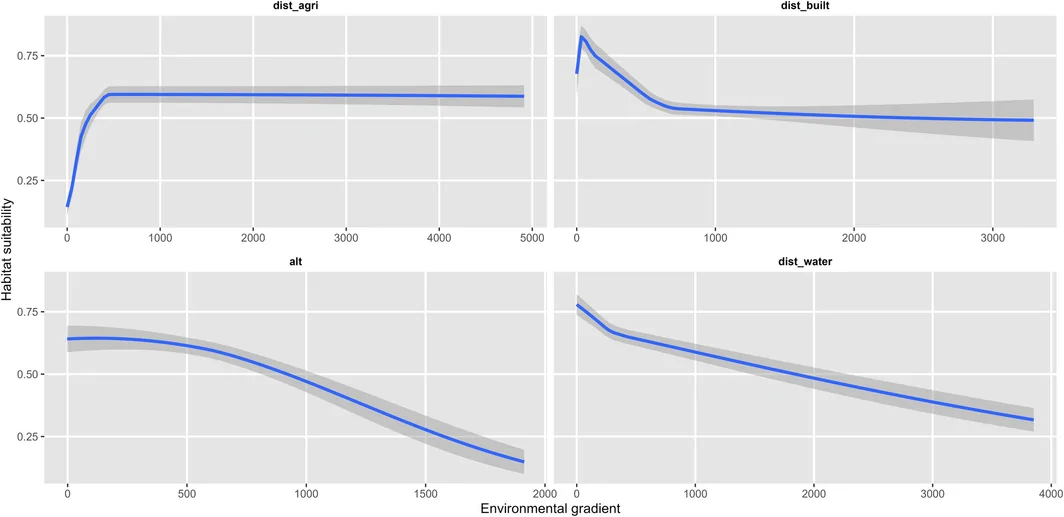
Response curve for the top four important variables for the distribution of wild boar.

### 3.3 Habitat Suitability Map

The potential distribution of the wild boar was estimated to cover 661.82 km^2^ representing 36.13% of Jeju Island in South Korea (Figure 4). Specifically, highly suitable habitat was spatially dispersed, accounting for 265.48 km^2^. Suitable habitat was patchy, accounting for 396.34 km^2^ of the study area (Table 2). The potential habitat overlayed 61.70% with forest, 27.91% with grassland, 3.70% with built‐up area, and so on (Table S5). The large proportion (more than 51%) of Hallasan National Park was estimated as potential habitat of wild boar (Figure 4).

**FIGURE 4:**
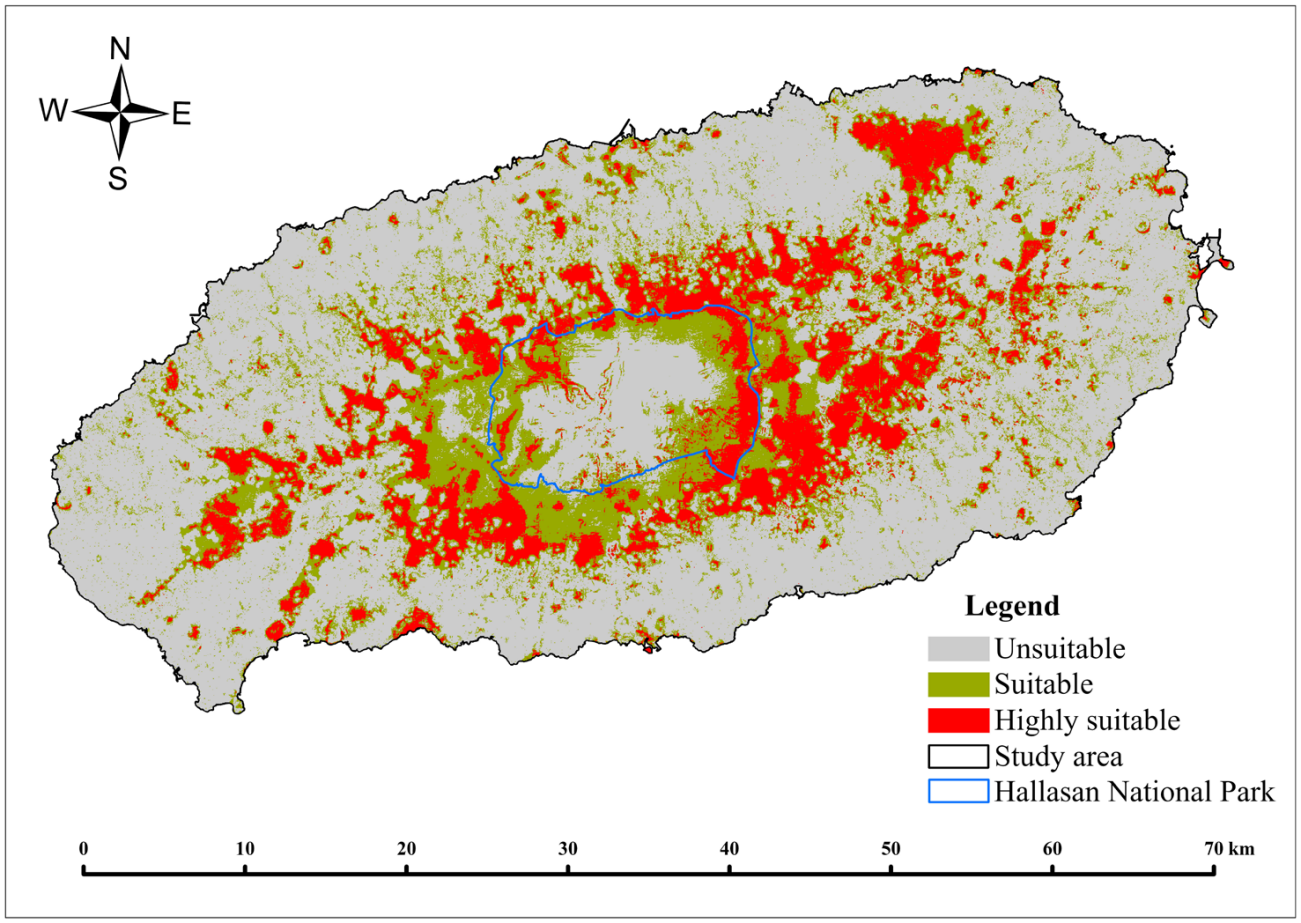
Predicted habitats of wild boar on Jeju Island, South Korea.

**TABLE 2:** Predicted habitat suitability of wild boar on Jeju Island.

S.N.	Category	Logistic probability of suitability	Area (km^2^)	Area (%)
1.	Unsuitable	0–0.38	1169.72	63.87
2.	Suitable	0.38–0.6	396.34	21.64
3.	Highly suitable	0.6–1.0	265.48	14.49
Total			1831.54	

### 3.4 Habitat Patch and Connectivity

Six habitat patches were identified for wild boar on Jeju Island, covering a combined area of 181.51km^2^. The average patch size was 30.25 ± 46.80 km^2^, where 68.06% overlapped with forest followed by 26.10% with grassland (Table S5). The largest habitat patch was located at Hallasan area (patch 1) with an area of 123.88 km^2^, whereas the smallest patch was situated at the Aewol area (Figure 5, Table 3).

**FIGURE 5:**
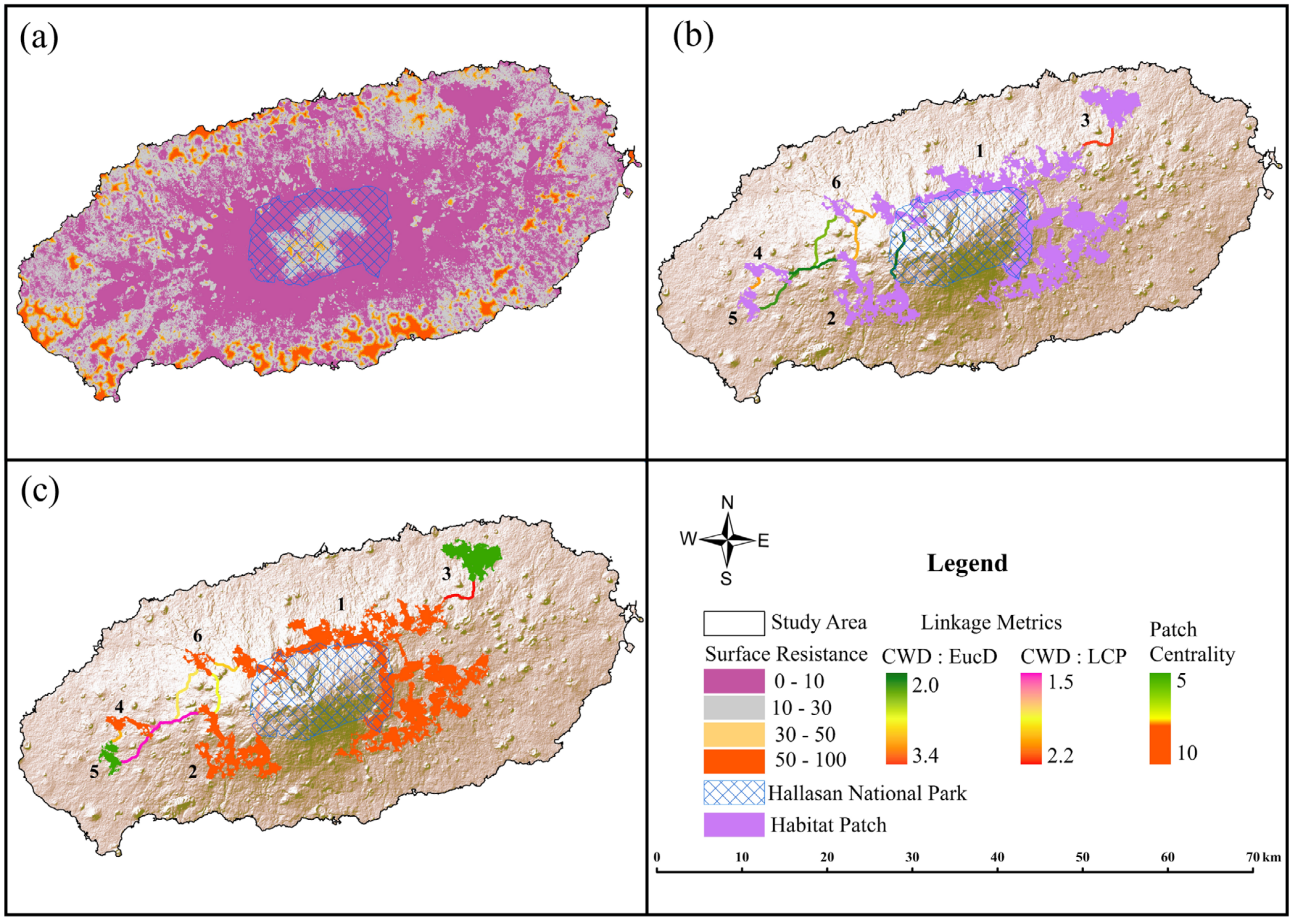
Landscape connectivity of wild boar on Jeju Island. (a) Surface resistance generated from habitat suitability model ranges from 0 to 100. (b) Habitat patch with ratio of cost‐weighted distance (CWD) to Euclidean distance of least‐cost path. (c) Centrality scores of core habitat patches with the ratio of cost‐weighted distance (CWD) to length of least‐cost patch (LCP).

**TABLE 3:** Habitat patches of wild boar on Jeju Island.

Patch code	Location	Area (Km^2^)
1	Hallasan area	123.88
2	Saekdal‐dong area	27.1
3	Manjanggul area	17.33
4	Geumakseo‐gil area	4.82
5	Gotjwal area	4.7
6	Aewol area	3.68

A total of eight linkages were identified among six habitat patches for wild boar across Jeju Island (Figure 5, Tables 3, and S4). The mean Euclidean distance (EucD) between patches was 4.70 km, while least‐cost path and cost‐weighted distance were 11.41 km and 6.48 km, respectively. The least‐cost pathways varied from 1.92 to 12.23 km with substantial variation in connectivity. The shortest linkage occurred from Geumakseo‐gil area (Patch 4) to Gotjwal area (Patch 5), while the longest was from Saekdal‐dong area (Patch 2) to Gotjwal area (Patch 5). The mean ratio of cost‐weighted distance to Euclidean distance and least‐cost path were 2.65 and 1.82, respectively. Connectivity was found to be the lowest between Manjanggul area (Patch 3) and Hallasan area (Patch 1), suggesting relatively high resistance, whereas the movement for wild boar in Saekdal‐dong area (Patch 2) area showed comparatively high accessibility (Figure 5; Tables 4, and S4). The Hallasan area and the Geumakseo‐gil area had the highest centrality score which were important habitat for maintaing overall connectivity within the landscape (Figure 5, Table S6).

**TABLE 4:** Characteristics of connectivity between habitat patches of wild boar on Jeju Island.

Category	Euclidean distance (EucD, km)	Cost‐weighted distance (CWD, km)	Least‐cost path (LCP, km)	CWD: EucD	CWD: LCP
Mean	4.70	11.41	6.48	2.65	1.82
SD	2.79	5.57	3.53	0.58	0.20
Range	7.52	16.06	10.27	1.48	0.61
Minimum	1.21	3.77	1.92	1.96	1.54
maximum	8.73	19.83	12.23	3.44	2.15

## 4 Discussion

The integrated assessment of the habitat suitability and landscape‐level connectivity provides the potential spatial dynamics of the wild boar on Jeju Island, South Korea. The final MaxEnt model demonstrated excellent predictive performance supported by higher AUC, TSS, and CBI scores. The results provide an ecological basis for identifying areas of potential habitat and connectivity for invasive species that may require management attention, thereby supporting prioritization of management intervention to limit and mitigate their potential impacts (Kunwar et al. 2024; Gao et al. 2025).

Incorporating fine‐scale environmental variables provides a detailed assessment of habitat suitability and environmental factors associated with wild boar distribution on Jeju Island, South Korea. This approach enhances the capacity to develop targeted and efficient management strategies for controlling invasive species (Perry et al. 2017). The final MaxEnt model identified four key environmental variables shaping the fine‐scale habitat suitability: distance to agriculture, distance to built‐up area, elevation, and distance to water. Among these, distance to agriculture was the strongest predictor, hightlighting the abundance and easy accessibility of forage resources in agricultural landscape, which may increase the occurrence of the species and increase the potential risk of human‐wild boar conflict (Milda et al. 2023). Similarly, the relatively higher suitability near built‐up area may indicate behavioral adaptability to anthropogenic landscapes and increased exposure to potential human‐wild boar conflict (Stillfried et al. 2017; Yıldız et al. 2026). Wild boar prefered coastal regions (0–500 m a.s.l.), particularly along with moderately rugged ridges (TRI: 0.15–0.50), suggesting that topographic conditions may influence the spatial distribution of wild boar (Bueno et al. 2009). Wild boar prefered to inhabit areas near forests, reflecting the importance of forests as sources of forage, shelter, and thermal refuge (Rutten et al. 2019; Jin et al. 2021; Lee, Kim, et al. 2024). Likewise, the habitat suitability decreases exponentially as the distance to water increases, further suggesting an association between wild boar and nearby inland water resources (Cordeiro et al. 2018). Suitability was also associated with open water (MNDWI > 0.5), potentially reflecting the important of inland water systems for thermoregulation and hydration (Cuevas et al. 2013). Additionally, higher suitability was in dense vegetation (NDVI: 0.6), which likely provides abundant food and hide (Gao et al. 2025). In contrast, variables such as distance to bare land and slope contributed relatively less to the model and might be interpreted cautiously.

The final Maxent model detected that suitable habitats for wild boar on Jeju Island were extensive and predominantely distributed in the mountainous region on Jeju Island. Forest, grassland and built‐up area were identified as suitable habitats for wild boar, reflecting ecological plasticity and the capacity to exploit both natural and human‐modified environments (Johann et al. 2020). Notably, a substantial proportion of Hallasan National Park was projected as a suitable habitat of wild boar, raising significant conservation concerns (Jin et al. 2021).

Integrating multiple least‐cost paths, we identified potential dispersal corridors across the island within the dispersal distance (Truvé and Lemel 2003), suggesting corridors effectively support dispersal and migration. The Saekdal‐dong area represented the most permeable lanscape for wild boar, whereas connectivity toward the Manjanggul area appears comparatively restricted, likely due to higher landscape resistance. Notably, the corridor connecting the Hallasan area and Saekdal‐dong area emerged as the most predictive corridor, traversing mountainous forest area. This pattern was consistent with the response curves, which demonstrated a strong preference for forest area. Such areas likely provide cover, abundant forage, and reduced human disturbance, thereby facilitating safer and energy‐efficient movement (Lewis et al. 2017). Similar connectivity patterns have been reported in the landscape dominated by forest, agricultural areas, and water resources (Tang et al. 2023; Kramer et al. 2024). The Hallasan area and the Saekdal‐dong area were identified as the most important habitats, providing overall landscape connectivity for wild boar. The identification of key corridors and important habitats have important implications for proactive management and control statrategies of invasive species (Lee, Kim, et al. 2024). Prioritizing these areas for population monitoring and targeted removal efforts, along with the installation of strategic fencing, and habitat modification, could be implemented to limit further dispersal of wild boar while minimizing impacts on other organisms (Carpio et al. 2021; Laguna et al. 2022; Ferens et al. 2025; Shin et al. 2026). Similar management approaches has been implemented in region where wild boar population have expanded rapidly, with intervention focusing on high density populations, key movement corridors and habitats to reduce dispersal and recolonization (Vargas‐Amado et al. 2023). Fencing and population control have also been integrated into wild boar management in several European countries; however, their effectiveness depends on fence design, maintenance, landscape configuration, and the spatial scale of implementation (Laguna et al. 2022; EFSA et al. 2024).

Despite these effeorts to assess habitat suitabilty and connevetivity of wild boar, our model has some uncertainity. Occurrence records were spatially rarefied within a 30 × 30 m grid to match the resolution of environmental predictors; however, a larger thining distance could improve the independnce of occurrence records. Climatic variables were not incorporated into the model, which may limit its ability to fully capture environmental drivers influencing wild boar distribution (Gao et al. 2025). However, this study was conducted on an island at a fine spatial scale, where available climatic variables would have required substantial downscaling, potentially introducing additional uncertainity into the fine‐scale habitat suitability projection. Small and spatially isolated habitat patches were excluded from connectivity analysis although, they may act as potential stopover sites and enhance to overall connectivity (Han, Wang, et al. 2022). In addition, the independent model validation was not conducted, limiting the ability to assess the reliability of landscape‐level connectivity using independent presence data (Vimercati et al. 2024). The landscape‐level least‐cost model for wild boar on Jeju Island utilized resistance surface derived from the species distribution model. The least‐cost distance approaches are widely used to evaluate functional connectivity among habitat patches based on cumulative landscape resistance (Dutta et al. 2016). However, this approach identifies optimal pathways and may not fully capture connectivity where dispersal is influenced by stochastic ecological processes and dynamic landscapes (Kim et al. 2024). Therefore, future studies should integrate telemetry‐based movement data with connectivity analyses to provide a comprehensive assessment of landscape permeability and functional connectivity. We recommend future studies focus on long‐term population monitoring and genetic assessment of gene flow would further improving our understanding of dispersal dynamics, landscape connectivity and the potential ecological consequences of wild boar expansion on Jeju Island.

## 5 Conclusion

This study provides a comprehensive modeling of habitat suitability and landscape connectivity for an invasive wild boar on Jeju Island. The findings indicate that suitable habitats are extensive and predominantly concentrated in forest, grassland, and built‐up area, demonstrating ecological plasticity. Distance to agriculture, distance to built‐up area, elevation, and distance to water were key environmental variables shaping the species' potential distribution. Furthermore, landscape‐level connectivity analysis revealed how connected habitat patches may facilitate movement across the landscape. The study provides guidance for targeted field surveys, long‐term monitoring, and empirical validation while prioritizing conservation interventions. Overall, this study identified potential risk areas, prioritized targeted monitoring, and early detection to limit future expansion and impacts of invasive species on Jeju Island.

## Author Contributions

**Binod Kunwar:** conceptualization (lead), data curation (lead), formal analysis (lead), methodology (lead), validation (lead), visualization (lead), writing – original draft (lead), writing – review and editing (lead). **Suraj Baral:** conceptualization (equal), formal analysis (lead), methodology (lead), validation (equal), visualization (equal), writing – original draft (equal), writing – review and editing (lead). **Young‐Hun Jeong:** data curation (equal), funding acquisition (lead), investigation (equal), writing – review and editing (equal). **Hong‐Shik Oh:** conceptualization (lead), data curation (equal), formal analysis (equal), funding acquisition (equal), supervision (lead), writing – original draft (equal), writing – review and editing (equal).

## Funding

The authors have nothing to report.

## Conflicts of Interest

The authors declare no conflicts of interest.

## Supporting information


**Figure S1:** Spearman's Rank Correlation between final set of variables used for the wild boar. Correlated variables (|r| ≥ 0.70) were excluded for the final distribution model.
**Figure S2:** Optimization of features and regulation multiplier selection through ENMeval Package for the final distribution model.
**Figure S3:** Response curve for analyzed variables; dist_oreum: Euclidean distance to Oreum, dist_forest: Euclidean distance to forest, dist_agri: Euclidean distance to agriculture, dist_grass: Euclidean distance to grassland, dist_built: Euclidean distance to built‐up area, dist_water: Euclidean distance to water, slope, ndvi: normalized difference vegetation index, variety: Index of habitat heterogeneity, dist_bare: Euclidean distance to bare land, mndwi: mean normalized difference water index, and tri: terrain ruggedness index for the distribution of sika deer on Jeju Island.
**Table S1:** Georeferenced Points of *Sus scrofa* on Jeju Island.
**Table S2:** Environmental variables used for habitat suitability of wild boar on Jeju Island, South Korea.
**Table S3:** Relative importance of analyzed variables for the optimized model.
**Table S4:** Landscape‐level connectivity between habitat patches of wild boar on Jeju Island.
**Table S5:** Land use and land cover of Jeju Island, South Korea in 2024 and area of potential habitat and core habitat in different land use and land cover types.

## Data Availability

The data that support this study have been provided in Supporting Information.
