# Supplementary material for: Modeling the Invasion: Habitat Suitability and Landscape Connectivity of Wild Boar (Sus scrofa) on Jeju Island, South Korea
Source: Ecol Evol. 2026 Sep 30;16(10):e74398. doi: 10.1002/ece3.74398 (PMC13627970; doi:10.1002/ece3.74398)
Supplement: Supplementary file 1 [file ECE3-16-e74398-s001.docx]

**Modelling the Invasion: Habitat Suitability and Landscape Connectivity of Wild Boar (*Sus scrofa*) on Jeju Island, South Korea**

Binod Kunwar^1^, Suraj Baral^2^, Young-Hun Jeong^1^, and Hong-Shik Oh ^1,3^*

^1^Interdisciplinary Graduate Program in Advanced Convergence Technology and Science, Jeju National University, Jeju Special Self-Governing Province, 63243, Republic of Korea

^2^Herpetology section, Leibniz Institute for the Analysis of Biodiversity Change, Museum Koenig Bonn, Germany

^3^Faculty of Science Education, Jeju National University, Jeju Special Self-Governing Province, 63243, Republic of Korea

**Supplementary Information:**

**Table S1** Georeferenced Points of *Sus scrofa* on Jeju Island.

**Table S2** Environmental variables used for habitat suitability of wild boar on Jeju Island, South Korea.

**Figure S1** Spearman’s Rank Correlation between final set of variables used for the wild boar. Correlated variables (|r| ≥ 0.70) were excluded for the final distribution model.

**Figure S2**. Optimization of features and regulation multiplier selection through ENMeval Package for the final distribution model.

**Table S3** Relative importance of analyzed variables for the optimized model.

**Figure S3** Response curve for analyzed variables; dist_oreum: Euclidean distance to Oreum, dist_forest: Euclidean distance to forest, dist_agri: Euclidean distance to agriculture, dist_grass: Euclidean distance to grassland, dist_built: Euclidean distance to built-up area, dist_water: Euclidean distance to water, slope, ndvi: normalized difference vegetation index, variety : Index of habitat heterogeneity, dist_bare: Euclidean distance to bare land, mndwi: mean normalized difference water index, and tri: terrain ruggedness index for the distribution of sika deer on Jeju Island.

**Table S4.** Landscape-level connectivity between habitat patches of wild boar on Jeju Island.

**Table S5.** Land use and land cover of Jeju Island, South Korea in 2024 and area of potential habitat and core habitat in different land use and land cover types.

**Table S1** Georeferenced Points of *Sus scrofa* on Jeju Island.

| **S.N.** | **Species** | **Longitude (°)** | **Latitude (°)** | **Remarks** |
| --- | --- | --- | --- | --- |
| 1 | *Sus scrofa* | 126.6034 | 33.3814 | Camera trap |
| 2 | *Sus scrofa* | 126.6008 | 33.3798 | Camera trap |
| 3 | *Sus scrofa* | 126.5836 | 33.3786 | Camera trap |
| 4 | *Sus scrofa* | 126.5842 | 33.3762 | Camera trap |
| 5 | *Sus scrofa* | 126.5951 | 33.3754 | Camera trap |
| 6 | *Sus scrofa* | 126.5602 | 33.4362 | Camera trap |
| 7 | *Sus scrofa* | 126.5588 | 33.4382 | Opportunistic sighting |
| 8 | *Sus scrofa* | 126.4626 | 33.3978 | Camera trap |
| 9 | *Sus scrofa* | 126.4536 | 33.3959 | Camera trap |
| 10 | *Sus scrofa* | 126.4524 | 33.3945 | Camera trap |
| 11 | *Sus scrofa* | 126.4604 | 33.3966 | Opportunistic sighting |
| 12 | *Sus scrofa* | 126.6507 | 33.3968 | Camera trap |
| 13 | *Sus scrofa* | 126.6491 | 33.3957 | Camera trap |
| 14 | *Sus scrofa* | 126.6457 | 33.3977 | Camera trap |
| 15 | *Sus scrofa* | 126.6483 | 33.399 | Camera trap |
| 16 | *Sus scrofa* | 126.4485 | 33.3645 | Camera trap |
| 17 | *Sus scrofa* | 126.4483 | 33.3713 | Camera trap |
| 18 | *Sus scrofa* | 126.4393 | 33.3666 | Camera trap |
| 19 | *Sus scrofa* | 126.3996 | 33.3766 | Camera trap |
| 20 | *Sus scrofa* | 126.4643 | 33.3589 | Camera trap |
| 21 | *Sus scrofa* | 126.3877 | 33.3778 | Camera trap |
| 22 | *Sus scrofa* | 126.4228 | 33.3797 | Camera trap |
| 23 | *Sus scrofa* | 126.4216 | 33.3833 | Camera trap |
| 24 | *Sus scrofa* | 126.4352 | 33.3856 | Camera trap |
| 25 | *Sus scrofa* | 126.454 | 33.3876 | Camera trap |
| 26 | *Sus scrofa* | 126.6075 | 33.4072 | Camera trap |
| 27 | *Sus scrofa* | 126.6102 | 33.4078 | Camera trap |
| 28 | *Sus scrofa* | 126.6202 | 33.406 | Camera trap |
| 29 | *Sus scrofa* | 126.6928 | 33.3679 | Camera trap |
| 30 | *Sus scrofa* | 126.6362 | 33.4362 | Camera trap |
| 31 | *Sus scrofa* | 126.6377 | 33.4374 | Camera trap |
| 32 | *Sus scrofa* | 126.4259 | 33.433 | Camera trap |
| 33 | *Sus scrofa* | 126.4847 | 33.414 | Camera trap |
| 34 | *Sus scrofa* | 126.4806 | 33.4129 | Camera trap |
| 35 | *Sus scrofa* | 126.4731 | 33.3732 | Camera trap |
| 36 | *Sus scrofa* | 126.4703 | 33.3794 | Opportunistic sighting |
| 37 | *Sus scrofa* | 126.4999 | 33.4198 | Camera trap |
| 38 | *Sus scrofa* | 126.4996 | 33.4177 | Camera trap |
| 39 | *Sus scrofa* | 126.5758 | 33.4349 | Opportunistic sighting |
| 40 | *Sus scrofa* | 126.6039 | 33.4304 | Camera trap |
| 41 | *Sus scrofa* | 126.5192 | 33.2965 | Camera trap |
| 42 | *Sus scrofa* | 126.524 | 33.3004 | Camera trap |
| 43 | *Sus scrofa* | 126.6819 | 33.3716 | Camera trap |
| 44 | *Sus scrofa* | 126.6835 | 33.3738 | Camera trap |
| 45 | *Sus scrofa* | 126.6411 | 33.3288 | Camera trap |
| 46 | *Sus scrofa* | 126.6353 | 33.3298 | Camera trap |
| 47 | *Sus scrofa* | 126.6694 | 33.3341 | Camera trap |
| 48 | *Sus scrofa* | 126.6692 | 33.3299 | Camera trap |
| 49 | *Sus scrofa* | 126.6721 | 33.3305 | Camera trap |
| 50 | *Sus scrofa* | 126.6568 | 33.3272 | Camera trap |
| 51 | *Sus scrofa* | 126.6498 | 33.3329 | Camera trap |
| 52 | *Sus scrofa* | 126.6489 | 33.3347 | Camera trap |
| 53 | *Sus scrofa* | 126.6529 | 33.3353 | Camera trap |
| 54 | *Sus scrofa* | 126.6284 | 33.327 | Camera trap |
| 55 | *Sus scrofa* | 126.6421 | 33.3253 | Opportunistic sighting |
| 56 | *Sus scrofa* | 126.6927 | 33.3638 | Camera trap |
| 57 | *Sus scrofa* | 126.6974 | 33.3629 | Camera trap |
| 58 | *Sus scrofa* | 126.6959 | 33.3571 | Camera trap |
| 59 | *Sus scrofa* | 126.6918 | 33.3599 | Camera trap |
| 60 | *Sus scrofa* | 126.5141 | 33.2655 | Camera trap |
| 61 | *Sus scrofa* | 126.5403 | 33.2461 | Opportunistic sighting |
| 62 | *Sus scrofa* | 126.566 | 33.3104 | Camera trap |
| 63 | *Sus scrofa* | 126.5632 | 33.3093 | Camera trap |
| 64 | *Sus scrofa* | 126.5671 | 33.3072 | Camera trap |
| 65 | *Sus scrofa* | 126.3387 | 33.2847 | Camera trap |
| 66 | *Sus scrofa* | 126.29 | 33.3321 | Camera trap |
| 67 | *Sus scrofa* | 126.2869 | 33.3313 | Opportunistic sighting |
| 68 | *Sus scrofa* | 126.6336 | 33.3167 | Camera trap |
| 69 | *Sus scrofa* | 126.654 | 33.33 | Camera trap |
| 70 | *Sus scrofa* | 126.6014 | 33.4247 | Camera trap |
| 71 | *Sus scrofa* | 126.6198 | 33.4134 | Camera trap |
| 72 | *Sus scrofa* | 126.6536 | 33.342 | Camera trap |
| 73 | *Sus scrofa* | 126.6603 | 33.3433 | Camera trap |
| 74 | *Sus scrofa* | 126.6669 | 33.3479 | Camera trap |
| 75 | *Sus scrofa* | 126.647 | 33.3388 | Camera trap |
| 76 | *Sus scrofa* | 126.6781 | 33.3688 | Camera trap |
| 77 | *Sus scrofa* | 126.6713 | 33.3753 | Camera trap |
| 78 | *Sus scrofa* | 126.6893 | 33.3965 | Camera trap |
| 79 | *Sus scrofa* | 126.6737 | 33.3998 | Camera trap |
| 80 | *Sus scrofa* | 126.6678 | 33.3904 | Camera trap |
| 81 | *Sus scrofa* | 126.626 | 33.4036 | Camera trap |
| 82 | *Sus scrofa* | 126.4857 | 33.4098 | Camera trap |
| 83 | *Sus scrofa* | 126.4777 | 33.4018 | Camera trap |
| 84 | *Sus scrofa* | 126.426 | 33.358 | Camera trap |
| 85 | *Sus scrofa* | 126.4937 | 33.4067 | Camera trap |
| 86 | *Sus scrofa* | 126.5134 | 33.4494 | Camera trap |
| 87 | *Sus scrofa* | 126.3891 | 33.3504 | Camera trap |
| 88 | *Sus scrofa* | 126.3941 | 33.3507 | Camera trap |
| 89 | *Sus scrofa* | 126.3971 | 33.3511 | Opportunistic sighting |
| 90 | *Sus scrofa* | 126.3837 | 33.3489 | Opportunistic sighting |
| 91 | *Sus scrofa* | 126.378 | 33.3664 | Camera trap |
| 92 | *Sus scrofa* | 126.3828 | 33.3663 | Camera trap |
| 93 | *Sus scrofa* | 126.6434 | 33.4339 | Camera trap |
| 94 | *Sus scrofa* | 126.6339 | 33.429 | Camera trap |

**Table S2** Environmental variables used for habitat suitability of wild boar on Jeju Island, South Korea.

| **S.N.** | **Code** | **Variable Description** | **Unit** | **Resolution (m)** | **Source** |
| --- | --- | --- | --- | --- | --- |
| 1. | LULC2024 | Land use landcover of 2024 | m | 1 | https://egis.me.go.kr/ |
| 2. | dist_built | Euclidean distance to built-up Area | m | 30 | Land use and land cover of 2024 (https://egis.me.go.kr/) |
| 3. | dist_agri | Euclidean distance to agriculture | m | 30 | Land use and land cover of 2024 (https://egis.me.go.kr/) |
| 4. | dist_forest | Euclidean distance to forest | m | 30 | Land use and land cover of 2024 (https://egis.me.go.kr/) |
| 5. | dist_grass | Euclidean distance to grassland | m | 30 | Land use and land cover of 2024 (https://egis.me.go.kr/) |
| 6. | dist_bare | Euclidean distance to barren land | m | 30 | Land use and land cover of 2024 (https://egis.me.go.kr/) |
| 7. | dist_water | Euclidean distance to water resources | m | 30 | Land use and land cover of 2024 (https://egis.me.go.kr/) |
| 8. | alt | Elevation above sea level | m | 30 | https://www.earthdata.nasa.gov/ |
| 9. | slope | Gradient of slope | ˚ | 30 | Digital Elevation Model |
| 10. | tri | Topographic heterogeneity | - | 30 | Digital Elevation Model |
| 11. | ndvi | Near infrared bands and red bands used | - | 30 | https://earthengine.google.com/ |
| 12. | ndbi | NIR and SWIR bands used | - | 30 | https://earthengine.google.com/ |
| 13. | mndwi | Green and SWIR bands used | - | 30 | https://earthengine.google.com/ |
| 14. | variety | Total number of habitat variables in 33 × 33 moving windows | - | 30 | Land use and land cover of 2024 (https://egis.me.go.kr/) |

**Fig S1.** Spearman’s Rank Correlation between final set of variables used for the wild boar. Correlated variables (|r| ≥ 0.70) were excluded for the final distribution model.

**Figure S2.** Optimization of feature class and regulation multiplier selection through ENMeval Package for the final distribution model.

**Table S3** Relative importance of analyzed variables for the optimized model.

| **Variable code** | **Description** | **Relative Importance** |
| --- | --- | --- |
| dist_agri | Euclidean distance to agriculture | 0.64 |
| dist_built | Euclidean distance to built-up area | 0.22 |
| alt | Elevation | 0.14 |
| dist_water | Euclidean distance to water | 0.13 |
| mndwi | Mean Normalized Difference Water Index | 0.11 |
| slope | Slope | 0.06 |
| dist_forest | Euclidean distance to forest | 0.04 |
| tri | Terrain Ruggedness Index | 0.02 |
| ndvi | Normalized Difference vegetation Index | 0.01 |
| dist_bare | Euclidean distance to bare land | 0.01 |

**Fig. S3.** Response curve for analyzed variables; dist_agri: Euclidean distance to agriculture, dist_built: Euclidean distance to built-up area, alt: elevation, dist_water: Euclidean distance to water, mndwi: mean normalized difference water index, slope, dist_forest: Euclidean distance to forest, tri: terrain ruggedness index, ndvi: normalized difference vegetation index, and dist_bare: Euclidean distance to bare land in the final MaxEnt Model for distribution of wild boar on Jeju Island.

**Table S4** Landscape-level connectivity between habitat patches of wild boar on Jeju Island.

| **S.N.** | **From the patch** | **To**  **the patch** | **Euclidean distance**  **(EucD, km)** | **Cost-weighted Distance (CWD, km)** | **Least-cost path (LCP, km)** | **CWD:**  **EucD** | **CWD:**  **LCP** |
| --- | --- | --- | --- | --- | --- | --- | --- |
| 1 | 3 | 1 | 3.51 | 12.07 | 5.61 | **3.44** | **2.15** |
| 2 | 1 | 6 | 2.04 | 6.24 | 3.23 | 3.06 | 1.93 |
| 3 | 1 | 2 | 5.5 | 10.77 | 6.34 | **1.96** | 1.70 |
| 4 | 6 | 2 | 3.15 | 9.62 | 5.2 | 3.05 | 1.85 |
| 5 | 6 | 4 | 8.5 | 19.83 | 11.01 | 2.33 | 1.80 |
| 6 | 2 | 4 | 4.96 | 10.11 | 6.32 | 2.04 | 1.60 |
| 7 | 2 | 5 | 8.73 | **18.85** | **12.23** | 2.16 | **1.54** |
| 8 | 4 | 5 | 1.21 | **3.77** | **1.92** | 3.12 | 1.96 |

**Table S5.** Land use and land cover of Jeju Island, South Korea in 2024 and area of potential habitat and core habitat in different land use and land cover types.

| **Land use and land cover class** | **Unsuitable habitat (km^2^)** | **Suitable habitat**  **(km^2^)** | **Highly Suitable habitat (km^2^)** | **Potential habitat (km^2^)** | **Potential habitat (%)** | **Habitat patches**  **(km^2^)** | **Habitat patches (%)** |
| --- | --- | --- | --- | --- | --- | --- | --- |
| Built-up area | 143.74 | 17.71 | 6.81 | 24.52 | 3.70 | 4.86 | 2.68 |
| Agriculture | 627.39 | 18.12 | 1.97 | 20.09 | 3.04 | 1.17 | 0.65 |
| Forest | 205.06 | 245.29 | 163.00 | 408.29 | 61.69 | 123.54 | 68.06 |
| Grassland | 159.63 | 100.01 | 84.71 | 184.72 | 27.91 | 47.38 | 26.10 |
| Bare land | 30.75 | 10.10 | 5.42 | 15.52 | 2.35 | 2.93 | 1.61 |
| Water | 3.15 | 5.11 | 3.57 | 8.68 | 1.31 | 1.64 | 0.90 |
| **Total** | 169.72 | 396.34 | 265.48 | 661.82 | 100 | 181.51 | 100 |

**Table S6** Centrality scores of habitat patches for wild boar on Jeju Island.

| **Patch code** | **Location** | **Centrality** |
| --- | --- | --- |
| 1 | Hallasan area | 9.90 |
| 2 | Saekdal-dong area | 9.06 |
| 3 | Manjanggul area | 5.00 |
| 4 | Geumakseo-gil area | 9.51 |
| 5 | Gotjwal area | 5.00 |
| 6 | Aewol area | 8.63 |
